# On demand treatment and home therapy of hereditary angioedema in Germany - the Frankfurt experience

**DOI:** 10.1186/1710-1492-6-21

**Published:** 2010-07-28

**Authors:** Emel Aygören-Pürsün, Inmaculada Martinez-Saguer, Eva Rusicke, Thomas Klingebiel, Wolfhart Kreuz

**Affiliations:** 1 Centre of Pediatrics III, Department of Hematology, Oncology and Hemostasis, Comprehensive Care Centre for Thrombosis and Hemostasis, Johann Wolfgang Goethe University Hospital, Frankfurt am Main, Germany

## Abstract

**Background:**

Manifestation of acute edema in hereditary angioedema (HAE) is characterized by interindividual and intraindividual variability in symptom expression over time. Flexible therapy options are needed.

**Methods:**

We describe and report on the outcomes of the highly individualized approach to HAE therapy practiced at our HAE center in Frankfurt (Germany).

**Results:**

The HAE center at the Frankfurt University Hospital currently treats 450 adults with HAE or AAE and 107 pediatric HAE patients with highly individualized therapeutic approaches. 73.9% of the adult patients treat HAE attacks by on-demand therapy with pasteurized pd C1-INH concentrate, 9.8% use additional prophylaxis with attenuated androgens, 1% of the total patient population in Frankfurt has been treated with Icatibant up to now. In addition adult and selected pediatric patients with a high frequency of severe attacks are instructed to apply individual replacement therapy (IRT) with pasteurized pd C1-INH concentrate. Improvement on Quality of Life items was shown for these patients compared to previous long-term danazol prophylaxis. Home treatment of HAE patients was developed in the Frankfurt HAE center in line with experiences in hemophilia therapy and has so far been implemented over a period of 28 years. At present 248 (55%) of the adult patients and 26 (24%) of the pediatric patients are practicing home treatment either as on demand or IRT treatment.

**Conclusions:**

In conclusion, the individualized home therapies provided by our HAE center, aim to limit the disruption to normal daily activities that occurs for many HAE patients. Furthermore, we seek to optimize the economic burden of the disease while offering a maximum quality of life to our patients.

## Introduction

### On demand treatment of acute angioedema in HAE type I and II

Hereditary angioedema (HAE) is based on a hereditary, life-long deficiency of C1-esterase-inhibitor (C1-INH). Patients with HAE suffer from recurrent, localized, acute edema attacks that can affect any body location. Mainly affected are subcutaneous tissues or mucous membranes, the gastrointestinal tract, and the throat, the latter leading to potentially life-threatening laryngeal edema. Manifestation of acute edema in hereditary angioedema is characterized by interindividual and intraindividual variability in symptom expression over time. The onset of the next attack, its location and its severity are unpredictable. Treatment options adapted on the specific needs of the individual patient need to be implemented based on the type and frequency of HAE attacks and should be re-evaluated from time to time [1,2].

Worldwide, five different therapy options for on demand therapy of acute attacks based on three distinct pathophysiological approaches are currently under clinical investigation or already approved in different countries. For replacement of lacking or dysfunctional C1-INH, three different C1-INH concentrates - two plasma-derived (pd) and one expressed in transgenic rabbits - are available or under investigation. Antagonism of the bradykinin B2-receptor, which is supposed to largely convey the increase in vascular permeability leading to acute angioedema in HAE, via the B2-receptor antagonist Icatibant is an entirely different approach. Additionally, inhibition of kallikrein, the activator of high molecular weight kininogen (HMWK) and therefore promotor of bradykinin formation, is a further potential therapeutic alternative.

In Germany, current treatment options approved for therapy of acute angioedema in HAE-Type I and II patients comprise intravenous replacement therapy with a pasteurized pd C1-INH concentrate and subcutaneous injection of the bradykinin B2-receptor antagonist Icatibant. Clinical efficacy has been demonstrated in retrospective studies for pasteurized pd C1-INH concentrate [3-5] and in prospective studies for both substances [6,7].

The clinical safety of pasteurized pd C1-NH concentrate has been proven over the last 25 years in more than 500,000 administrations (data on file, CSL Behring), which were well tolerated. In this period of time, only 8 allergic or anaphylactic reactions, including four episodes in one HAE-Type I patient from Frankfurt, have been observed. This corresponds to 1:50,000 administrations and thus to classification of allergic reactions as a "very rare" (< 1:10,000) adverse event according to the CIOMS III standard categories for classification of adverse drug reaction frequency [8]. No proven cases of viral transmission from pasteurized pd C1-INH concentrate have been shown in the last 25 years (data on file, CSL Behring) [9]. Pasteurized pd C1-INH concentrate can be used in pediatric and adult patients. It is well tolerated also in pregnant and breast-feeding women [10].

Clinical experience with Icatibant is limited up to now, as it has been only recently (2008) approved for HAE in Germany. The safety of Icatibant has been shown in clinical studies [11]. The most frequent side effect, encountered in almost all treated subjects, is a transient, itchy, and sometimes painful erythema at the subcutaneous injection site. Common adverse reactions of Icatibant are nausea, abdominal pain, asthenia, increased blood creatinine phosphokinase, abnormal liver function test, dizziness, headache, nasal congestion and rash. Icatibant may be used up to 3 times within 24 hours. In the elderly patient (> 65 years) experience with Icatibant is limited, as less than 5% of the study population belongs to this age group [11]. Moreover, clearance of Icatibant may decline with age, which may lead to a higher exposure in elderly patients (> 75 yrs.). Presently, no experience with administration of Icatibant in pregnant or breastfeeding women or in children exists. Caution should be observed in the administration of Icatibant to patients with acute ischemic heart disease or unstable angina pectoris and to patients in the weeks following a stroke. According to the product's prescribing information, no dose adjustment with hepatic or renal impairment is required.

### Differential therapy of C1-INH deficiency at the Frankfurt HAE Center

Our HAE center at Frankfurt University Hospital currently treats 450 adults with hereditary or acquired (AAE) C1-INH deficiency (430 HAE and 20 AAE patients), and 107 pediatric HAE patients with highly individualized therapeutic approaches.

The majority (73.7%) of adult patients at the center are candidates for sole on-demand therapy with pasteurized pd C1-INH concentrate of acute HAE attacks. However, 9.8% of adult patients qualify for long-term prophylaxis with attenuated androgens (e.g., danazol), although the latter, while being widely-used worldwide, is not licensed for use in HAE [2]. Potential break-through attacks in attenuated androgen-treated patients are treated using pasteurized pd C1-INH concentrate on demand.

In addition, we provide the option of individual replacement therapy (IRT) with pd C1-INH concentrate for high-risk patients [2]. HAE patients affected by a high frequency of severe attacks (> 1 per week), and unresponsive to long-term prophylaxis with attenuated androgens were advised to administer pasteurized pd C1-INH concentrate on early signs of an acute attack. Patients usually administer a dose of 500 to 1000 U of pasteurized pd C1-INH at each early sign of attack (i.e. up to twice a week). This therapy protocol is being supported by the favorable pharmacokinetic properties of a long half-life of pasteurized pd C1-INH concentrate in different HAE patient subgroups (see Table 1) [12]. With IRT, a significant reduction of annual attack rates compared to previous danazol prophylaxis was seen. Particularly, laryngeal attacks were entirely abolished. Moreover, a significant efficacy of IRT on all items of quality of life (QoL) investigated was verified [2]. Ongoing long-term studies will furthermore evaluate the efficacy and safety of IRT.

**Table 1:** Pharmacokinetics of pasteurized pd C1-INH Concentrate (median values)

	Incremental IVR [%rise/U/kg bw]	Tmax [hr]	T 1/2 [hr]	MRT [hr]	AUC [U*hr/mL]	Clearance [mL/kg*hr]	Vss [mL/kg]
Children on Demand n = 6	2.2	0.6	32.9	47.5	13.4	1.1	50.0
Adults on Demand n = 19	2.0	1.0	39.1	56.5	16.7	0.9	56.5
Adults IRT n = 15	2.9*	0.5	30.9**	44.6**	15.7	1.0	37.5***

*p = 0.006, ** p = 0.052, *** p = 0.004, statistically significant differences of Adults IRT vs. Adults on demand

IVR = in vivo recovery

Tmax = Median time to maximum functional pd C1-INH level

T1/2 = Terminal elimination half-life

MRT = Mean residence time

AUC = area under the curve

Vss = Volume of distribution at steady state

Home therapy with pd C1-INH concentrate in HAE patients was developed in line with hemophilia therapy, where the missing protein is administered by the patient at home on an as-needed basis. Home treatment with plasma derived and recombinant factor VIII and factor IX concentrates is an established therapy in North America since 1975 [13] and is included in the German Guidelines on hemophilia therapy. In Frankfurt, home therapy with pd C1-INH concentrate for use in on demand treatment or within the IRT protocol has been implemented for a period of 28 years. At present, a total of 274 patients (49% of all patients with C1-INH deficiency treated by the center) are practicing home treatment. Out of these, 248 are adult patients (55% of adult patients) and 26 children (24% of pediatric patients). The age of the relevant patient groups ranges between 18-81 years and 6-17 years, respectively. In young pediatric patients, parents are the care-givers for home therapy. With pasteurized pd C1-INH home therapy, a rapid response to treatment is observed. There were no reports of treatment- related adverse events or life-threatening events.

The experience with Icatibant in Frankfurt is very limited at the moment. 1% of the total patient population at the Frankfurt HAE center has been treated with Icatibant within studies or in a routine setting. Due to its pathophysiologic mechanism, which is different from C1-INH, Icatibant is a valuable treatment option in two of our patients in whom pasteurized pd C1-INH concentrate cannot be applied: one patient with a known allergy to pd C1-INH concentrate and one patient with acquired angioedema non-responsive to pd C1-INH concentrate due to high anti-C1-INH antibody titers. In the therapy of a life-long condition, pharmacoeconomic considerations may also be of relevance. Based on the current prices in Germany, the cost of on demand treatment of an acute attack with one dose of Icatibant (30 mg) is equal to the cost of one dose of 20 U/kg body weight (bw) of pasteurized pd C1-INH concentrate for a 70 kg standard patient. For a 100 kg patient receiving a dose of 20 U/kg bw therapy with pasteurized pd C1-INH concentrate, is more expensive compared to Icatibant.

However, in all other settings, e.g. in 50 kg-, 70 kg- or 100 kg - patients treated with a dose of 10 U/kg pd C1-INH concentrate, or even in a 50 kg- patient treated with 20 U/kg pd C1-INH concentrate, treatment with a single dose of Icatibant is more expensive compared to pd C1-INH concentrate. This is important in view of the fact that in contrast to the results of the IMPACT-1 study [6] the necessity for a dose of 20 U/kg of pd C1-INH concentrate is rare in clinical practice. In the vast majority of cases, 500 - 1000 U pasteurized pd C1-INH concentrate, usually corresponding to ≤ 10 U/kg to < 20 U/kg, are effective for treatment of acute attacks, particularly when treatment is initiated rapidly [3-6]. Furthermore, follow-up doses of pd C1-INH concentrate can be administered in fractions of 500 U, which is significantly more economical compared to follow-up injections of Icatibant that may be commonly required [11].

In conclusion, the individualized therapeutic strategies provided by our HAE center aim to limit the constraints in daily life that occur for many HAE patients.

Furthermore, we seek to optimize the economic burden of the disease while offering a maximum quality of life to our patients.

## List of Abbreviations

AAE: acquired angioedema; bw: body weight; C1-INH: C1-esterase-inhibitor; HAE: hereditary angioedema; IRT: individual replacement therapy; pd: plasma derived

## Competing interests

This study was supported by an unrestricted grant from CSL Behring GmbH, Hattersheim, Germany. CSL Behring GmbH had no influence on the collection, analysis, or interpretation of data, and had no influence on the decision to submit this manuscript for publication.

The authors certify that they have no affiliation with or financial involvement in any organization or entity with direct financial interest in the subject matter or materials discussed in this manuscript (e.g., employment, consultancies, board membership, stock ownership). Any research or project support is identified in the manuscript. The corresponding author receives support for participation of scientific conferences from CSL Behring, Shire and Viropharma.

## Authors' contributions

All authors contributed equally to this manuscript and have read and approved the final version of this manuscript.
